# 

**Published:** 1898-07-30

**Authors:** 


					THE HOSPITAL. "The standard of highest purity."?Lancet, July 30th, 1898.
CADBURY'S ^ u igM CADBURY'S
COGOA
ABSOLUTELY PURE.
COCOA
NO DRUGS OR ALKALIES USED.
mWICH HOSBl-TAl
No. 618. Vol. XXIV. [XSJ Saturday,
Jtjly 30th, 1898.
L Bee p?300. J PRICE TWOPENCE.

				

